# Lithium levels and lifestyle in patients with bipolar disorder: a new tool for self-management

**DOI:** 10.1186/s40345-023-00291-x

**Published:** 2023-03-16

**Authors:** I. Zorrilla, S. Lopez-Zurbano, S. Alberich, I. Barbero, P. Lopez-Pena, E. García-Corres, J. P. Chart Pascual, J. M. Crespo, C. de Dios, V. Balanzá-Martínez, A. Gonzalez-Pinto

**Affiliations:** 1 Bioaraba Health Research Institute, Vitoria-Gasteiz, Spain; 2 Psychiatry Department, Osakidetza Basque Health Service, Araba University Hospital, Vitoria-Gasteiz, Spain; 3 University of the Basque Country UPV/EHU, Vitoria-Gasteiz, Spain; 4 Biomedical Research Networking Centre in Mental Health (CIBERSAM), Madrid, Spain; 5 Departament of Psychiatry, Bellvitge University Hospital, Bellvitge Biomedical Research Institute-Idibell, Barcelona, Spain; 6 Departament of Clinical Sciences, University of Barcelona, Bellvitge Campus, L´Hospitalet de Llobregat, Barcelona, Spain; 7 Psychiatric Department, University Hospital La Paz, IdiPAZ, Madrid, Spain; 8 Teaching Unit of Psychiatry and Psychological Medicine, Department of Medicine, University of Valencia, Valencia, Spain

**Keywords:** Bipolar disorder, Lifestyle factors, Healthy diet, Sleep, Lithium, Wearable

## Abstract

**Background:**

Patients should get actively involved in the management of their illness. The aim of this study was to assess the influence of lifestyle factors, including sleep, diet, and physical activity, on lithium levels in patients with bipolar disorder.

**Methods:**

A multicenter study was performed. In total, 157 lithium measurements were done biweekly in a sample of 65 patients (35 women) over 6 weeks. Lifestyle, based on total sleep hours and physical activity, was assessed by actigraphy. Diet was evaluated using the Mediterranean Lifestyle Index (Medlife).

**Results:**

35.4% of patients had a normal weight. The mean Medlife score was 14.5 (± 2.5) (moderate-good adherence to Mediterranean diet). BMI, daily dose of lithium and intensity of physical activity had a combined effect on lithium levels, after adjustment for other variables. Patients who practiced intense physical exercise, who took lower doses and had a higher BMI exhibited lower levels of lithium.

**Conclusions:**

Higher physical activity and BMI contribute to lower lithium levels. Patients should be made aware of these relationships to improve their perception of control and self-management. Lifestyle-based interventions contribute to establishing a more personalized medicine.

## Introduction

Bipolar disorder (BD) is a chronic episodic mood disorder. The age of onset is < 25 years in 75% of patients (González Pinto et al. 2009), with the disease causing excess mortality and a 10–20-year loss of life expectancy, mainly due to cardiovascular disease (McIntyre et al. 2020). This illness is related to high suicide rates (Gonzalez-Pinto et al. 2006) and is one of the main causes of incapacity for work among young people (Grande et al. 2016). Some of the needs of these patients are not being met, and new therapeutic approaches based on personalized medicine are required (Bauer et al. 2018).

Lithium is the gold-standard treatment for BD (Malhi et al. 2017) due to its mood stabilizing, anti-suicide (Gonzalez-Pinto et al. 2006), antimanic (Fountoulakis et al. 2022; Nivoli et al. 2012), and antidepressant (González-Pinto et al. 2018) effects. In addition, lithium improves the functionality of patients with bipolar disorder and alcohol or other substance abuse. Moreover, this agent seem to have protective effects against cognitive impairment (Fountoulakis et al. 2022).

Despite its efficacy, the use of lithium therapy is declining worldwide (Lin et al. 2020; Bohlken et al. 2020). Some reasons for this decline include the poor quality of the information provided to patients, the complexity of the therapy, as regular monitoring of blood levels of lithium is needed, and insufficient training of psychiatrists in the use of lithium therapy (Young and Hammond 2007; González-Pinto et al. 2021; Pérez de Mendiola et al. 2021), among other reasons. The narrow therapeutic range, the fact that it is excreted mostly via the kidneys, and the risk of toxicity requires close monitoring of clearance and specific training for use (Grandjean and Lithium 2009). Although there is consensus on the standard therapeutic range (0.6–1.2), the ideal therapeutic level has been reduced to 0.60–0.80 mmol/L. In case the patient shows good response, this range can be reduced to 0.40–0.60 mmol/L to minimize side effects (Nolen et al. 2019). Small changes in lithium levels have been associated with relapses. Severus et al. (2009), described lower levels (− 0.09) of lithium before manic relapses, with respect to depressive relapses. A review of studies assessing lithium levels associated an abrupt decrease of more than 0.2 mmol/L of serum levels with an increased risk of relapse (Severus et al. 2008).

To overcome these challenges, a more active involvement of patients in their treatment is essential (Bauer et al. 2016; Gomes et al. 2022). For this purpose, it is necessary that patients receive adequate information about their process, treatments, and factors under their control (González-Pinto et al. 2021; Sanada et al. 2016). In patients with bipolar disorder taking lithium salts, awareness of the effects of lifestyle on lithium levels will help them improve their sense of self-control. This will ultimately encourage them to get more involved in their treatment and improve their lifestyle. In fact, a sense of control, or an internal locus of control, has been proven to increase resilience against depression (Khumalo and Plattner 2019; Reynaert et al. 1995; Shara et al. 2018).

Whereas the influence of weight on lithium levels has been the subject of previous research (Grandjean and Lithium 2009), the influence of diet, sleep (Spano et al. 2022), and physical activity has been neglected. Sleep is one of the most important lifestyle factors in BD and its regularity is used as a therapeutic tool to improve prognosis (Morton et al. 2022). With respect to body size, weight is related to creatinine clearance, the most important factor related to lithium levels (ElDesoky et al. 2008; Methaneethorn and Sringam 2019). In relation to physical activity, in a previous experiment performed in four healthy controls who took lithium salts for seven days, strenuous exercise was found to be associated with decreased lithium levels (Jefferson et al. 1982).

Wearables are useful for monitoring lifestyle factors, especially physical activity and total sleep time (Bauer et al. 2020). In a recent study carried out with wearables in patients with bipolar disorder, two of the three factors that resulted from the measurements performed (sleep, activation but not communication) were useful for detecting manic symptoms (Ebner-Priemer et al. 2020).

The relationship between lifestyle factors and lithium levels has been scarcely studied. We hypothesized that lifestyle is related to lithium levels. This study was conducted to test our hypothesis. The aim of this research study was to assess the influence of lifestyle factors, including sleep, diet, BMI and physical activity, on lithium levels in subjects diagnosed with bipolar disorder.

## Methods

A multicenter study was performed in five Spanish University Hospitals. Patients diagnosed with bipolar disorder were recruited from the following centers: Hospital Universitario Araba, Hospital Universitario Bellvitge, Hospital Universitario la Paz, Hospital Universitari i Politècnic La Fe and Hospital Clínico Universitario de Valencia. All patients had been taking lithium salts for at least 2 years prior to inclusion, were adherent to treatment, and suffered from DSM-5-bipolar disorder (SCID) (First et al. 2016). Other criteria for inclusion were being 18–65 years old, speaking Spanish fluently, being able to handle a smartphone, and signing an informed consent form. Only the patients who attended at least a follow-up visit were included in the study. Exclusion criteria included the use of non-steroidal anti-inflammatory drugs (NSAIDs), antihypertensives and/or diuretics, and occurrence of a (hypo)manic or depressive episode during follow-up. Patients with renal impairment, high creatinine levels or eGFR < 60, patients with infections, fever or diarrhea were also excluded. Lithium levels in bipolar patients were measured at three time points. Six-week follow-up was performed. The study was approved by the Institutional Review Boards of the participating centers.

Clinical interviews were conducted to collect sociodemographic and clinical data, including sex, age, weight (kg), height (m), prescribed daily dose of lithium, and concomitant use of medication, alcohol, cannabis and/or other substances during the study. Body Mass Index was calculated as kg/m^2^ and analyzed as a continuous variable. Based on BMI, patients were categorized as with normal weight (18.5–24.9 kg/m^2^), overweight (25–29.9 kg/m^2^) or obese (≥ 30 kg/m^2^).

Lifestyle, assessed in terms of physical activity, was evaluated using an actigraph watch type LifeVit AT-510HR Series (http://www.lifevit.es/pulserahr.php) for 6 weeks. The watch was connected to a smartphone that automatically collected data and sent it to a centralized system. Outliers—like 0 min of sleep—were excluded. For this study, only total sleep and daily steps were considered. In follow-up visits, the individual mean of total sleep and daily steps during the previous 14 days were evaluated.

Diet was evaluated using the Mediterranean Lifestyle Index (MEDLIFE) (Sotos-Prieto et al. 2015, 2021). A higher score indicates good adherence to the Mediterranean diet.

The profile of patients was assessed at baseline using a set of scales. The study was explained to patients, and compliance with inclusion and exclusion criteria was assessed. Finally, informed consent was obtained. Functioning was evaluated using the Global Assessment of Functioning test (GAF) (Endicott et al. 1976) and the Functioning Assessment Short Test (FAST) (Rosa et al. 2007). The clinical status of patients was assessed on the Young Mania Rating Scale (YMRS) (Young et al. 1978) and the Hamilton Depression Rating Scale (HAM-D-21) (Hamilton 1967). Finally, quality of life was evaluated using the Spanish version of the World Health Organization Quality of Life Assessment-Bref (Lucas-Carrasco 2012).

**Fig. 1:**
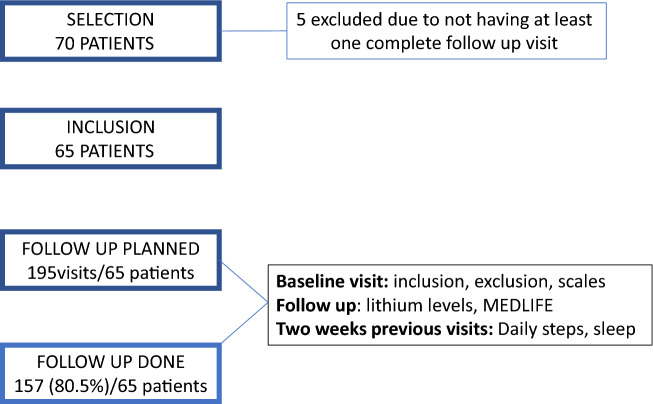
Description of the study

Blood was drawn to measure lithium levels at each bi-weekly visit (Fig. 1) for 6 weeks. Sociodemographic, anthropometric, clinical, and functional variables were recorded in each visit. For this purpose, patients were asked to attend an appointment in their center of reference in fasting conditions. Blood was drawn at 8:30 a.m., 12 h after the last dose of lithium.

The study was approved by the Ethical Committee of the Hospital Universitario de Alava (Spain), in accordance with the Declaration of Helsinki (Expte. 2019-047).

### Statistical analysis

Sample size was calculated based on a power of 80% to detect a significance level of 5%, assuming an equivalence limit of 0.15. Theoretical values correspond to the limits of the interval in relation to lithium levels in blood: [0.6 mEq/L, 1.2 mEq/L]; standard error was 0.40. A total of 63 patients needed to be included. Considering a dropout rate of 10%, 70 patients were included. Sample size was calculated using Ene 3.0.

For statistical analysis, clinical and lifestyle data for the previous 2 weeks, as recorded in each biweekly visit, were considered to assess their association with lithium levels. Descriptive statistics were used to describe sociodemographic and clinical characteristics (mean, standard deviation (± SD), frequencies, percentages). We used linear mixed-effects models for the analysis of repeated measures. For that purpose, backward elimination regression was used to find the model that best explained the individual effect of lifestyle variables on lithium levels. Clinical and actigraphy data related to lifestyle (total sleep time, diet, BMI, intensity of physical activity according to daily steps, dose, use of alcohol and drugs) of the previous 2 weeks were considered for analysis. These models were adjusted for sex and age to eliminate potential confounding effects. Models were also adjusted for the effect of time, considering the longitudinal design of the study. They included a random effect to account for the repeated measure structure of data and were fitted using maximum likelihood techniques assuming normality of error terms. Diagnosis of the models was performed to determine whether the underlying normality assumptions held. All statistical analyses were performed with the statistical package SPSS.23 and R 3.1.2.

## Results

A total of 157 measurements (80.5% of the planned measurements) of lithium levels were performed in 65 outpatients over 6 weeks. Baseline sociodemographic, clinical, and functional characteristics of the sample are summarized in Table 1. Table 2 describes lifestyle-related factors. The mean age of patients was 45.62 (± 11.53) years and 53.8% were female. According to the three BMI categories, patients were divided into normal BMI (35.4%), overweight (38.5%), and obese (26.2%). The mean daily dose of lithium prescribed was 975.4 mg (± 253.7), and mean lithium level was 0.694 (± 0.145). As many as 40% of patients performed light physical activity (< 5000 daily steps), 33.8% moderate physical activity (between 5000–10.000 daily steps), and 26.2% practiced intense physical activity (> 10.000 daily steps). Further data are shown in Table 2.

**Table 1:** Sociodemographic, clinical, and functional characteristics of the sample at baseline

Variable	Mean/frequency	SD/percentage
Sex		
Male	30	46,2%
Female	35	53,8%
Age	45.62	11.53
BMI		
Normal weight	23	35.4%
Overweight	25	38.5%
Obesity	17	26.2%
Total	28.17	5.07
YMRS	2.22	4.90
HAM-D-21	6.39	6.14
WHOQOL		
Physical health	22.39	5.04
Psychological	17.87	6.17
Social relationships	8.48	2.76
Environment	27.74	5.66
FAST		
Autonomy	2.23	3.47
Occupational functioning	5.74	6.14
Cognitive functioning	4.95	4.33
Financial issues	1.26	1.96
Interpersonal relationships	4.39	4.98
Leisure time	1.89	2.11
Total	20.42	17.94
GAF	74.11	15.43

**Table 2:** Lifestyle-related variables

Variable	Mean/frequency	SD/percentage
Alcohol use	10	15.4%
Drugs use	5	7.7%
Medlife	14.42	2.52
Daily steps	7304.85	4077.65
Sleep duration baseline	8.758	3.405

*MEDLIFE* Mediterranean Lifestyle Index

### Lifestyle-related factors related to lithium levels

**Fig. 2:**
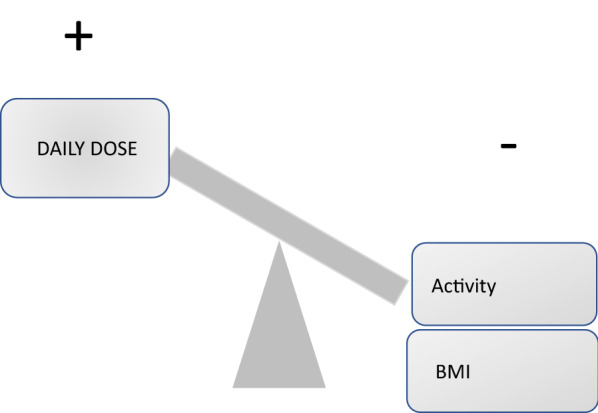
Positive and negative relationships with lithium levels

The final result of the linear mixed-effects model is presented in Table 3. BMI, daily physical activity, and daily dose of lithium were found to have a significant impact on lithium levels (Fig. 2). A higher BMI (considered as continuous variable), higher physical activity, and a lower dose of lithium reduced lithium levels significantly. Regarding BMI, the same results were obtained when the grouping variable was included (normal weight, overweight and obesity) instead of the continuous variable. Hence, patients with a higher BMI (≥ 30 kg) had lower lithium levels, as compared to those with normal weight (18.5–24.9 kg) (b = − 0.10234, p = 0.015). The confounding variable age was also significant, with younger patients showing lower lithium levels. Although time did not have significant effects on lithium levels (b = 0.00825, p = 0.439), it was maintained in the model to adjust for the longitudinal nature of the study. Neither following a Mediterranean diet, total sleep time, nor substance use (alcohol and drugs) had any effect on lithium levels (Mediterranean diet: b = 0.00263, p = 0.807; total sleep time: b = 0.000001, p = 0.992; alcohol: b = − 0.03825, p = 0.230; drugs: b = 0.09202, p = 0.071).

**Table 3:** Linear mixed-effects model of lifestyle-related factors on lithium levels

Variable	b coefficient	*p*-value
Age	0.00312	0.027
BMI	− 0.00819	0.011
Daily steps	− 0.03772	0.033
Daily dose of lithium	0.00021	< 0.001

With respect to the amount of influence in lithium levels, increasing daily steps from 5000 to 15,000, there is a decrease in lithium levels of 0.05 mEq/L. The same is seen when decreasing the dose of lithium in 200 mg. A decrease of 2 points of BMI had a smaller effect on lithium levels (0.03 mEq/L).

## Discussion

The main finding of this study is that it shows the relationship between physical activity and blood levels of lithium. Some variables have been identified in the literature as significant predictors of lithium levels, the most important being lithium dose (Abou-Auda et al. 2008; Hsu et al. 2021). However, although physical activity is known to play a significant role in bipolar disorder, this is the first study to assess and demonstrate its effects on lithium levels. This association is important, especially if we consider the relevance given to lifestyle factors in recommendations for patients with bipolar disorder.

The results of this study reveal that physical activity, measured based on daily steps, are negatively associated with lithium levels, after controlling for lithium dose. According to our results, an increase of 10,000 daily steps reduces lithium levels by 0.05 mEq/L, which is equivalent to a 200 mg-reduction of lithium salt dose. Reverse causality hardly explains this association, as lithium levels were related to physical activity in the previous 2 weeks. The reason why lithium levels decrease with physical activity is unknown, although some authors have hypothesized that lithium loss in sweat may be involved. Jefferson et al. conducted an experiment in young healthy controls treated with lithium, who were included in an intensive exercise training plan. Although subjects became dehydrated during the race, their serum lithium levels decreased (Jefferson et al. 1982). Lithium loss was higher than sodium loss. Such high increases of activity are rare in patients with bipolar disorder in remission. Nevertheless, activation is a core symptom of mania and hypomania (Bauer et al. 2020; Arrasate et al. 2014); therefore, controlling daily steps may help patients being aware of their mental state and activation. In addition, lithium levels have been documented to decrease prior to a manic relapse (Severus et al. 2009). According to our results, this can be related to an increase in activity and daily steps.

Although changes in daily steps are generally small in most stable patients, subsyndromal symptoms may be accompanied by higher changes in the number of steps and, consequently, result in small reductions/increases in lithium levels. In general, we strongly recommend patients to increase physical activity and daily steps to be healthier. To avoid abrupt changes in lithium levels, activity should be increased progressively. Alternatively, if a patient is planning to increase physical activity significantly, it is recommended that lithium levels are previously measured. Moreover, increasing the number of daily steps could be useful during subsyndromal depressive episodes, as activation has positive effects on depression and reduces lithium levels. According to Severus et al. (2009, 2008), during depressive episodes, there is an increased risk of reducing physical activity and having higher levels of lithium. In addition, being aware of these small changes in lithium levels can help avoid the side effects of fluctuating levels of lithium. According to Gitlin (2016), side effects are more frequent in patients with depressive symptoms. Diarrhea and polyuria are more frequent when lithium levels increase. One of the strategies recommended for managing these subjective and objective side effects is reducing the dose (Gitlin 2016). Based on our results, we alternatively recommend promoting activation. This solution is good for improving depressive symptoms (Walsh et al. 2022), reducing lithium levels slightly, and improving the cardiovascular system (Falkai et al. 2022). Physical activity is effective and should be promoted among bipolar patients to prevent cardiovascular disease, which is one of the targets of a healthy lifestyle (Bauer et al. 2016; García et al. 2020).

BMI also had a significant inverse influence in lithium levels. According to our results, measuring lithium levels is recommended when there is a rapid increase/decrease of BMI, although a long time is generally needed to lose or gain 1 point of body mass index. In line with our results, Abou-Auda et al. (Sotos-Prieto et al. 2015) reported that lithium levels are influenced by weight and daily dose, among other factors. In addition Sproule et al. (2000) and Tondo et al. (2017) observed that lithium clearance is higher in obese subjects; therefore, a higher dose is needed for these patients to obtain the same therapeutic effect. The effect of weight on lithium levels was first studied by Zetin et al. (1986), who identified weight and concomitant prescription of tricyclic antidepressants, as well as age and sex, as important variables to be considered for estimating the optimal lithium dose using an algorithm. We did not find any association between lithium levels and total sleep time.

The influence of diet and nutrition on lithium levels has been scarcely studied in patients with bipolar disorder treated with lithium salts. Although low-sodium diets have been associated with elevated lithium levels, the influence of dietary patterns and healthy diet has not yet been studied (Grandjean and Lithium 2009). In our study, Mediterranean diet did not have any effect on lithium levels. In a previous study conducted in the general population, lithium levels (general population not treated with lithium salts) were associated with higher intakes of leafy vegetables, root vegetables and fruits and lower intakes of pasta, rice, pork, chocolate, sweets, soft drinks, sauces and snacks, (Enderle et al. 2020); however, there are no previous studies on patients receiving lithium treatment. Nevertheless, a healthy diet has a positive impact on the prognosis of bipolar disorder. The rate of MEDLIFE in our sample was of 14.50. These results correspond to median values of the third quartile (moderate-good adherence to Mediterranean lifestyle) of the ENRICA cohort, conducted in more than 11,000 subjects older than 18 years and representative of the general population in Spain (ElDesoky et al. 2008).

Our cohort of patients with BD had a relatively good lifestyle, as they were quite adherent to the Mediterranean diet and performed moderate physical activity daily (7304.85 steps). Nevertheless, the rates of overweight and obesity were high. Therefore, there is room for improvement and a healthier lifestyle should be recommended. In addition, it is important to consider that abrupt changes in lifestyle are common in patients with BD, due to subsyndromal symptoms and relapses, which can influence lithium levels, thereby contributing to a higher risk of destabilization.

Although lifestyle factors are critical in patients with BD, a percentage of these patients have an unhealthy lifestyle, including poor diet and sleep patterns, sedentary habits, and tobacco and alcohol/substance use (Firth et al. 2019). In fact, there is an increasing awareness that changes in lifestyle may be effective in the prevention and treatment of BD (Firth et al. 2020). Preliminary evidence supports that specific lifestyle-based interventions may improve clinical outcomes and cognition in mood disorders (Severus et al. 2009; Brietzke et al. 2018; Miskowiak et al. 2022). Future studies should be performed to replicate our findings and examine whether these strategies modify lithium levels and are useful for the optimization and personalization of lithium prescriptions. Strategies for managing their disease should be explained to patients with a diagnosis of BD (Nicholas et al. 2017). This approach would contribute to a more personalized management of mental illness, and provide the opportunity to implement timely assessments and interventions (Bell et al. 2017).

This study has some limitations, the most important being that it is a naturalistic research study. We did not measure other biological factors related to lithium levels, such as renal function, although all patients were within the normal range of renal function. Another limitation is that the effect of diet was measured with MEDLIFE scale. This scale primarily assess diet, but it also includes six items of Mediterranean lifestyle habits unrelated to diet. Furthermore, we cannot exclude that significant relationships are mediated by factors that have not yet been investigated.

The study also has several strengths. We controlled for other variables and designed a multicenter study to limit bias and increase external validity. Daily activity data was obtained in the previous 2 weeks. Our findings await replication in studies in other countries. In addition, the sample size is quite limited but similar to others used in previous studies, such as the one by Abu-Auda (Bauer et al. 2020), who performed 137 measurements of lithium levels.

In conclusion, physical activity and BMI are related to lithium levels. Thus, patients taking lithium should be aware that physical activity reduces lithium levels, and that a low BMI is associated with higher lithium levels at the same prescribed dose. As previously reported (Blixen et al. 2016), the success of a comprehensive treatment to manage bipolar disorder relies on patient’s perception of control, support, clear information, and self-management. As stated by Conn and Ruppar (2017), healthcare professionals should use lifestyle-based interventions to implement a more personalized medicine. This study, along with lifestyle psychiatry studies provide patients with useful tools to increase their internal locus of control and adherence to treatment.

## Data Availability

Data used in this study are available upon request.
